# High percent body fat mass predicts lower risk of cardiac events in patients with heart failure: an explanation of the obesity paradox

**DOI:** 10.1186/s12877-020-01950-9

**Published:** 2021-01-06

**Authors:** Katsuhiko Ohori, Toshiyuki Yano, Satoshi Katano, Hidemichi Kouzu, Suguru Honma, Kanako Shimomura, Takuya Inoue, Yuhei Takamura, Ryohei Nagaoka, Masayuki Koyama, Nobutaka Nagano, Takefumi Fujito, Ryo Nishikawa, Tomoyuki Ishigo, Ayako Watanabe, Akiyoshi Hashimoto, Tetsuji Miura

**Affiliations:** 1 Department of Cardiovascular, Renal and Metabolic Medicine, Sapporo Medical University School of Medicine, South-1, West-16, Chuo-ku, Sapporo, 060-8543 Japan; 2 Department of Cardiology, Hokkaido Cardiovascular Hospital, Sapporo, Japan; 3 Division of Rehabilitation, Sapporo Medical University Hospital, Sapporo, Japan; 4 Department of Public Health, Sapporo Medical University School of Medicine, Sapporo, Japan; 5 Division of Hospital Pharmacy, Sapporo Medical University Hospital, Sapporo, Japan; 6 Division of Nursing, Sapporo Medical University Hospital, Sapporo, Japan; 7 Division of Health Care Administration and Management, Sapporo Medical University School of Medicine, Sapporo, Japan

**Keywords:** Heart failure, Body mass index, Obesity, Skeletal muscle, Fat

## Abstract

**Background:**

Although high body mass index (BMI) is a risk factor of heart failure (HF), HF patients with a higher BMI had a lower mortality rate than that in HF patients with normal or lower BMI, a phenomenon that has been termed the “obesity paradox”. However, the relationship between body composition, i.e., fat or muscle mass, and clinical outcome in HF remains unclear.

**Methods:**

We retrospectively analyzed data for 198 consecutive HF patients (76 years of age; males, 49%). Patients who were admitted to our institute for diagnosis and management of HF and received a dual-energy X-ray absorptiometry scan were included regardless of left ventricular ejection fraction (LVEF) categories. Muscle wasting was defined as appendicular skeletal muscle mass index < 7.0 kg/m^2^ in males and < 5.4 kg/m^2^ in females. Increased percent body fat mass (increased FM) was defined as percent body fat > 25% in males and > 30% in females.

**Results:**

The median age of the patients was 76 years (interquartile range [IQR], 67–82 years) and 49% of them were male. The median LVEF was 47% (IQR, 33–63%) and 33% of the patients had heart failure with reduced ejection fraction. Increased FM and muscle wasting were observed in 58 and 67% of the enrolled patients, respectively. During a 180-day follow-up period, 32 patients (16%) had cardiac events defined as cardiac death or readmission by worsening HF or arrhythmia. Kaplan-Meier survival curves showed that patients with increased FM had a lower cardiac event rate than did patients without increased FM (11.4% vs. 22.6%, *p* = 0.03). Kaplan-Meier curves of cardiac event rates did not differ between patients with and those without muscle wasting (16.5% vs. 15.4%, *p* = 0.93). In multivariate Cox regression analyses, increased FM was independently associated with lower cardiac event rates (hazard ratio: 0.45, 95% confidence interval: 0.22–0.93) after adjustment for age, sex, diabetes, muscle wasting, and renal function.

**Conclusions:**

High percent body fat mass is associated with lower risk of short-term cardiac events in HF patients.

**Supplementary Information:**

The online version contains supplementary material available at 10.1186/s12877-020-01950-9.

## Background

Body mass index (BMI) is an easily measurable and quantitative anthropometric indicator of body mass and nutritional status, and it is widely used for the definition and classification of obesity. It has been established that obesity, generally defined as a BMI ≥ 25 kg/m^2^, is an independent risk factor of incident chronic diseases including hypertension, type 2 diabetes mellitus, cancer, and heart failure (HF) [1–3]. However, it has been shown that mortality is not proportionally increased with BMI-defined obesity. Results from epidemiological studies suggest that overweight (BMI, 25.0–29.9 kg/m^2^) or class I obesity (BMI, 30–34.9 kg/m^2^) is not associated with a worse clinical outcome or is associated even with a favorable outcome in the study population [4–6]. That is the case in HF patients; HF patients with a higher BMI had a lower mortality rate than that in HF patients with normal or lower BMI, a phenomenon that has been termed the “obesity paradox” [2, 3, 7–10]. In addition, a U-shaped relationship between BMI and mortality has been reported for both patients with heart failure with reduced ejection fraction (HFrEF) and patients with heart failure with preserved ejection fraction (HFpEF) [9, 10]. However, the reason for this complex relationship between BMI and mortality in HF patients has not been fully elucidated.

Cachexia is a hallmark in the advanced stage of chronic diseases including HF and is defined as involuntary loss of at least 5% of non-edematous body weight [11]. A pioneering study in this field by Anker et al. showed that the presence of cachexia is an independent predictor for mortality even after adjustment for age, exercise capacity, and severity of HF [12]. Body composition analyses by the use of dual-energy X-ray absorptiometry (DEXA) revealed that cachexic patients had reduced fat mass, reduced lean mass (muscle mass), and reduced bone mineral content [13]. Thus, a plausible explanation of the obesity paradox is that high BMI increases the risk of HF development, while HF-induced cachexia per se is associated with increased mortality. However, the natural history of each composition of the body (i.e., fat, muscle mass, bone mineral content) during progression of HF and the impact of the composition on prognosis of HF patients remain to be elucidated. In the present study, we used a DEXA scan, the best technique for analyzing body composition in research studies [14], to examine the relationships between fat and muscle masses and clinical outcome in HF patients.

## Methods

This study was approved by the Clinical Investigation Ethics Committee of Sapporo Medical University Hospital (Number 302–104). This study was carried out by the opt-out method of our hospital website. Informed consent was obtained in the form of opt-out on the website.

### Study subjects

This study was conducted in strict adherence with the principles of the Declaration of Helsinki. This study was a single center, retrospective, and observational study. Consecutive patients who were admitted to our institute for diagnosis and management of HF during the period from January 1, 2016 to October 31, 2018 were retrospectively enrolled. HF was diagnosed according to Japanese Circulation Society/Japanese Heart Failure Society Guidelines for Heart Failure [15]. Patients who were admitted for acute decompensation heart failure were also included. DEXA measurements were performed after their symptoms were relieved to New York Heart Association (NYHA) functional class III in patients who had NYHA functional class IV symptoms at the time of admission. We excluded patients who had in-hospital death and were transferred to other hospitals at the time of discharge from our hospital. For patients who underwent multiple DEXA measurements for assessment of body composition during hospitalization, the last data set was used for analysis.

### DEXA measurements

Body composition analyses were performed as previously reported [16]. Whole and regional fat/lean masses of patients were analyzed by using the Horizon DXA System (HOLOGIC, Waltham, MA, USA) and lean mass was defined as an index of muscle mass. Increased percent body fat mass (increased FM) was defined as DEXA-measured percent body fat mass > 25% in males and > 30% in females according to the results of a Japanese epidemiological study showing the association of percent body fat mass with prevalence rates of cardiac and metabolic diseases [17]. Appendicular skeletal muscle mass (ASM) was calculated as the sum of bone-free lean masses in the arms and legs. ASM index (ASMI) was defined as ASM/height^2^. The cut-off values of ASMI for muscle wasting were < 7.00 kg/m^2^ in males and < 5.40 kg/m^2^ in females according to the criteria of the Asian Work Group for Sarcopenia [18].

### Laboratory data and echocardiography

Measurement of laboratory data (serum albumin, hemoglobin, creatinine, estimated glomerular filtration rate [eGFR], fasting plasma glucose, insulin, total cholesterol, low density lipoprotein cholesterol, high density lipoprotein cholesterol, triglyceride and N-terminal pro B-type natriuretic peptide [NT-proBNP]) and echocardiographic analyses were performed as previously reported [16]. The left ventricular ejection fraction (LVEF) was measured by the modified Simpson method.

### Clinical endpoint

A cardiac event was defined as cardiac death or unscheduled readmission by worsening HF or arrhythmia. Data for clinical endpoints during a period of 180 days after hospital discharge in the patients were collected from the medical records. Intervals between outpatient clinic visits were 4 ~ 8 weeks depending on the patients.

### Statistical analysis

Data are presented as means ± standard deviation, medians (interquartile range [IQR]), or percentages for variables. Differences in continuous variables between patients with and those without increased FM or muscle wasting were tested by the chi-square test or the Mann-Whitney U test. Differences in categorical variables between patients with and those without increased FM or muscle wasting were examined by the chi-square test. Survival curves were calculated by the Kaplan-Meier method, and statistical significance of differences between the curves was assessed by log-rank statistics. Univariate and multivariate Cox proportional hazard models were used to determine the contribution of increased FM or muscle wasting to cardiac event rates. A probability value of < 0.05 was adopted as the critical level of statistical significance. Statistical analysis was performed using R version 3.5.2 (R Foundation for Statistical Computing, Vienna, Austria).

## Results

### Baseline characteristics

Of 244 patients screened, 198 patients met inclusion criteria without exclusion criteria and contributed to the analyses. Baseline clinical characteristics of the patients are shown in Table 1. The median age of the patients was 76 years (IQR, 67–82 years) and 49% of them were male. The median BMI of the patients was 21.8 kg/m^2^. Forty-four percent of the patients were classified as NYHA functional class III. The median left ventricular ejection fraction (LVEF) was 47% (IQR, 33–63%) and 33% of the patients had HFrEF. Hypertension, dyslipidemia, diabetes and chronic kidney disease (CKD) were present in 70, 52, 38 and 50% of the patients, respectively. The most frequent etiology of HF was cardiomyopathy (37%), followed by valvular heart disease (29%) and ischemic heart disease (19%).

**Table 1:** Patient’s characteristics

Parameters	Overall		Increased FM					Muscle wasting				
			Absent		Present		*p* value	Absent		Present		p value
	*n* = 198		*n* = 84 (42%)		*n* = 114 (58%)			*n* = 65 (33%)		*n* = 133 (67%)		
Age, yrs.	76	[67–82]	75	[67–81]	76	[68–82]	0.489	78	[67–83]	74	[67–81]	0.119
Male, n (%)	102	(52)	48	(57)	54	(47)	0.224	30	(46)	72	(54)	0.366
Height, cm	158	±10	159	±10	157	±10	0.370	157	±11	158	±9	0.320
Weight, kg	53	[47–63]	49	[43–56]	57	[51–67]	< 0.001	63	[51–70]	51	[44–58]	< 0.001
BMI, kg/m^2^	22	[20–24]	20	[18–22]	23	[22–26]	< 0.001	25	[23–28]	20	[19–23]	< 0.001
NYHA-FC III, n (%)	87	(44)	44	(52)	43	(38)	0.056	17	(26)	70	(53)	0.001
LVEF, %	47	[33–63]	47	[31–62]	48	[34–64]	0.342	55	[36–64]	47	[32–62]	0.237
< 40%, n (%)	66	(33)	32	(38)	34	(30)	0.286	19	(29)	47	(35)	0.487
eGFR, ml/min/1.73cm^2^	57	[38–77]	55	[34–80]	58	[41–76]	0.724	56	[45–76]	59	[34–78]	0.997
**DEXA data**												
ASMI, kg/m^2^	5.6	[5.0–6.5]	5.5	[4.9–6.3]	5.8	[5.0–6.6]	0.205	7.0	[5.8–7.5]	5.1	[4.7–5.9]	< 0.001
PBF, %	28.3	[23.6–34.0]	22.8	[19.6–24.8]	33.1	[29.2–36.4]	< 0.001	30.0	[25.5–35.1]	27.1	[23.0–33.6]	0.025
**Comorbidity**												
Hypertension	138	(70)	51	(61)	87	(76)	0.027	47	(72)	91	(68)	0.693
Dyslipidemia	103	(52)	32	(38)	71	(62)	0.001	37	(57)	66	(50)	0.416
Diabetes	76	(38)	34	(41)	42	(37)	0.710	16	(25)	60	(45)	0.009
CKD	98	(50)	42	(50)	56	(49)	1.000	31	(48)	67	(50)	0.839
**Medication**												
ACE-I or ARB	91	(46)	33	(39)	58	(51)	0.141	34	(52)	57	(43)	0.271
Beta blocker	134	(68)	58	(69)	76	(67)	0.841	44	(68)	90	(68)	1.000
Loop diuretics	122	(62)	61	(73)	61	(54)	0.010	34	(52)	88	(66)	0.084
MRA	92	(47)	44	(52)	48	(42)	0.198	22	(34)	70	(53)	0.019
**Etiology**							0.636					0.011
Cardiomyopathy	73	(37)	31	(37)	42	(37)		27	(42)	46	(35)	
VHD	58	(29)	21	(25)	37	(33)		24	(37)	34	(26)	
IHD	38	(19)	18	(21)	20	(18)		4	(6)	34	(26)	
Others	29	(15)	14	(17)	15	(13)		10	(15)	19	(14)	

*FM* fat mass, *BMI* body mass index, *NYHA-FC* New York heart association-functional class, *LVEF* left ventricular ejection fraction

*eGFR* estimated glomerular filtration ratio, *DEXA* dual-energy X-ray absorptiometry, *ASMI* appendicular skeletal muscle mass index, *PBF* percent body fat, *CKD* chronic kidney disease, *ACE-I* angiotensin-converting-enzyme inhibitor, *ARB* angiotensin II receptor blocker;

*MRA* mineralocorticoid receptor antagonist, *VHD* valvular heart disease, *IHD* ischemic heart disease

Increased percent body fat mass (increased FM) was defined as percent body fat > 25% in males and > 30% in females

Muscle wasting, i.e., reduction in skeletal muscle mass, was defined as appendicular skeletal muscle mass index < 7.0 kg/m^2^ in males and < 5.4 kg/m^2^ in females

**Fig. 1:**
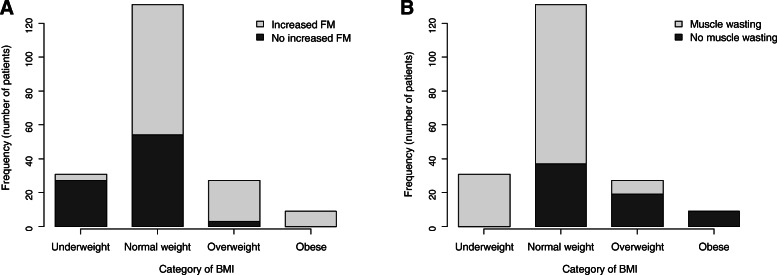
Proportions of patients with increased body fat mass and patients with muscle wasting in body mass index-based obesity classifications. Body mass index (BMI)-based definition of obesity established by the World Health Organization is as follows: Underweight: BMI < 18.5, Normal weight: 18.5≦BMI < 25, Overweight: 25≦BMI < 30, Obese: 30≦BMI. Increased percent body fat mass (increased FM) was defined as dual-energy X-ray absorptiometry-measured percent body fat mass > 25% in males and > 30% in females. Muscle wasting was defined by the cut-off values of appendicular skeletal muscle mass index: < 7.00 kg/m^2^ in males and < 5.40 kg/m^2^ in females

Increased FM and muscle wasting were observed in 58 and 67% of the enrolled patients, respectively. As shown in Table 1, there was no significant difference in age or gender between patients with and those without increased FM or muscle wasting. As expected, BMI level was higher in patients with increased FM than in those without increased FM, and patients with muscle wasting had lower BMI than did patients without muscle wasting. The proportion of patients with increased FM (Fig. 1a) and the proportion of patients with muscle wasting (Fig. 1b) in each BMI-based obesity categories are shown in Fig. 1. This figure clearly indicates that the presence of increased FM or muscle wasting in each patient cannot be predicted by BMI alone.

### Comparison of groups with and without increased FM

In patients with increased FM, NYHA functional class III symptoms tended to be less frequent (38% vs 52%), the proportion of patients with hypertension (76% vs. 61%) and dyslipidemia (62% vs. 38%) were significantly higher, and loop diuretics (54% vs. 73%) were less frequently prescribed compared with those in patients without increased FM (Table 1). Plasma albumin and creatinine levels were similar in patients with and those without increased FM. However, plasma insulin and triglyceride levels were higher and levels of high density lipoprotein cholesterol and NT-proBNP were lower in patients with increased FM than in patients without increased FM (Table 2).

**Table 2:** Laboratory data and body composition analysis

Parameters	Overall		Increased FM					Muscle wasting				
			absent		present		p value	absent		present		p value
	n = 198		n = 84 (42%)		n = 114 (58%)			n = 65 (33%)		n = 133 (67%)		
Hemoglobin, g/dl	11.8	[10.4–13.3]	11.5	[10.1–13.2]	12.0	[11.0–13.4]	0.249	11.8	[10.5–13.5]	11.6	[10.4–13.2]	0.367
Albumin, g/dl	3.5	[3.3–3.8]	3.5	[3.2–3.7]	3.6	[3.3–3.9]	0.322	3.6	[3.4–3.8]	3.5	[3.2–3.8]	0.154
Creatinine, mg/dl	1.02	[0.76–1.50]	1.04	[0.74–1.60]	0.99	[0.81–1.33]	0.755	1.03	[0.82–1.22]	0.97	[0.75–1.57]	0.964
FPG, mg/dl	91	[83–102]	89	[80–102]	91	[84–102]	0.287	90	[86–97]	91	[82–105]	0.837
Insulin, μIU/ml	5.1	[3.4–7.6]	3.8	[2.4, 6.3]	5.9	[4.4, 9.4]	< 0.001	5.6	[4.1, 8.6]	4.7	[3.1, 7.2]	0.056
TC, mg/dl	162	[143–186]	160	[133–189]	163	[145–182]	0.358	161	[147–186]	162	[140–187]	0.806
HDL-C, mg/dl	51	[42–61]	52	[42–64]	50	[41–57]	0.055	48	[40–60]	52	[43–62]	0.363
LDL-C, mg/dl	90	[73–111]	87	[72–106]	92	[75–112]	0.218	90	[76–111]	90	[73–111]	0.692
TG, mg/dl	88	[64–121]	76	[59–103]	98	[77–123]	< 0.001	91	[70–123]	87	[64–115]	0.441
NT-proBNP, pg/dl	1522	[750–3240]	2316	[807–4130]	1236	[622–2566]	0.002	1133	[496–2654]	1673	[879–3581]	0.049

*FM* fat mass, *FPG* fasting plasma glucose, *TC* total cholesterol, *LDL-C* low density lipoprotein cholesterol, *HDL-C* high density lipoprotein cholesterol, *TG* triglyceride, *ASMI* appendicular skeletal muscle mass index; PBF, percent body fat

Increased percent body fat mass (increased FM) was defined as percent body fat > 25% in males and > 30% in females

Muscle wasting, i.e., reduction in skeletal muscle mass, was defined as appendicular skeletal muscle mass index < 7.0 kg/m^2^ in males and < 5.4 kg/m^2^ in females

### Comparison of groups with and without muscle wasting

In patients with muscle wasting, NYHA functional class III symptoms were more frequent (53% vs. 26%), and the proportion of patients with diabetes (45% vs. 25%) and patients on mineralocorticoid receptor antagonists (53% vs. 34%) were higher than those in patients without muscle wasting. Proportions of etiologies of HF were also different between patients with and without muscle wasting (Table 1). While plasma albumin and creatinine levels were similar in patients with and those without muscle wasting, plasma NT-proBNP level was significantly higher in patients with muscle wasting.

### Impacts of increased FM and muscle wasting on cardiac event rates

**Fig. 2:**
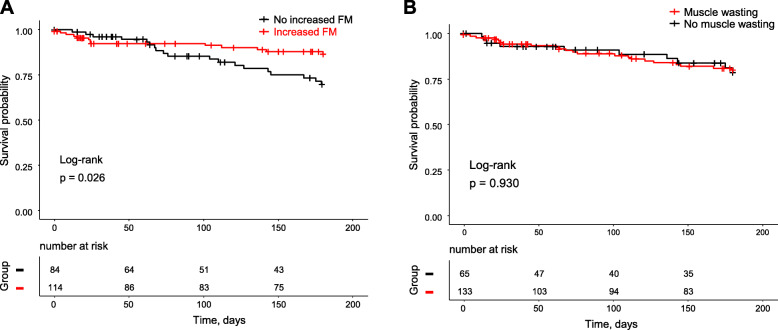
Kaplan-Meier event-free survival curves. **a** Group with increased percent body fat mass (red line) vs. group without increased body fat mass (black line). **b** Group with muscle wasting (red line) vs. group without muscle wasting (black line). Increased percent body fat mass was defined as dual-energy X-ray absorptiometry-measured percent body fat mass > 25% in males and > 30% in females. Muscle wasting was defined by the cut-off values of appendicular skeletal muscle mass index: < 7.00 kg/m^2^ in males and < 5.40 kg/m^2^ in females. FM, percent body fat mass

**Fig. 3:**
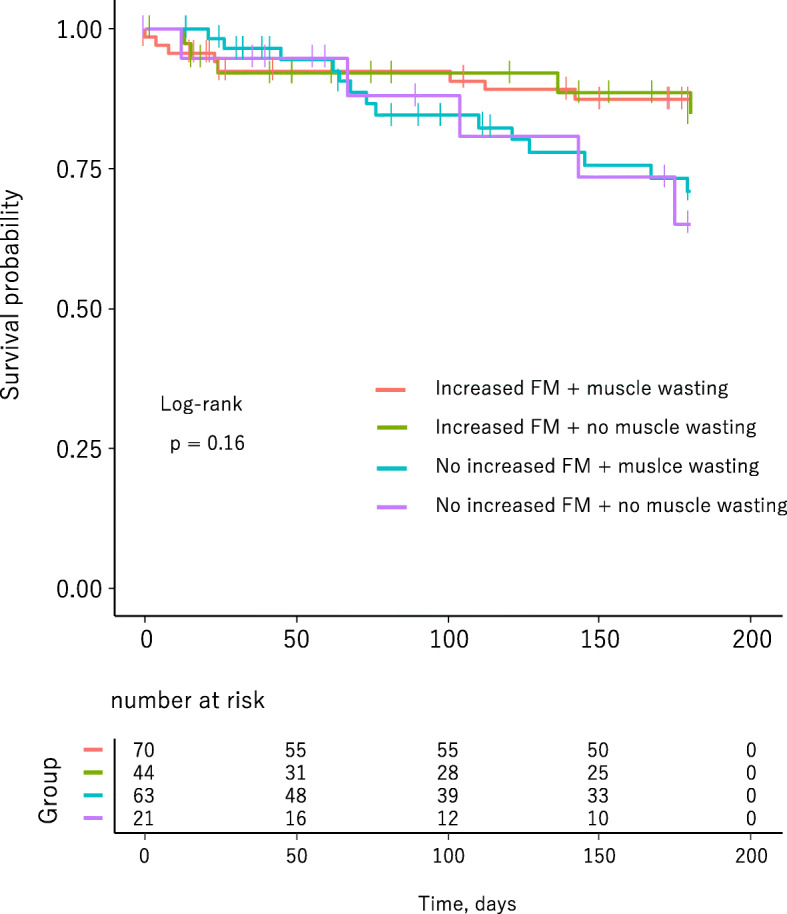
Kaplan-Meier event-free survival curves for each body composition category. Increased percent body fat mass was defined as dual-energy X-ray absorptiometry-measured percent body fat mass > 25% in males and > 30% in females. Muscle wasting was defined by the cut-off values of appendicular skeletal muscle mass index: < 7.00 kg/m^2^ in males and < 5.40 kg/m^2^ in females. FM, percent body fat mass

During a 180-day follow-up period, 32 patients (16%) had cardiac events. Kaplan-Meier survival curves showed that patients with increased FM had a significantly lower cardiac event rate than did patients without increased FM (11.4% vs. 22.6%, *p* = 0.03, Fig. 2a). On the other hand, there was no difference in cardiac event rates between patients with muscle wasting and those without muscle wasting (16.5% vs. 15.4%, *p* = 0.93, Fig. 2b). In addition, presence of muscle wasting had no effects on cardiac event rates also in HF patients with increased FM (Fig. 3). In multivariate Cox-proportional hazard analyses that were adjusted for age, sex, diabetes, and renal function, increased FM was independently associated with lower cardiac event rate (hazard ratio: 0.45, 95% confidence interval: 0.22–0.93, Table 3). The independent association between increased FM and cardiac event rate was lost by inclusion of NT-proBNP level and NYHA functional class III, but not LVEF, into the Cox-proportional hazard model (Supplementary Table 1). In contrast to increased FM, muscle wasting was not selected as an independent determinant of cardiac events in multivariate analysis (Table 3 and Supplementary Table 1).

**Table 3:** Univariate and Multivariate analyses by Cox-proportional hazards model

Parameters	Univariate model			Multivariate model								
				model 1			model 2			model 3		
	HR	95% CI	p value	HR	95% CI	p value	HR	95% CI	p value	HR	95% CI	p value
Age, yrs	1.003	(0.978–1.029)	0.808	1.001	(0.976–1.028)	0.912	0.990	(0.963–1.017)	0.460	0.993	(0.966–1.021)	0.616
Sex, male	0.848	(0.424–1.697)	0.641	0.852	(0.422–1.717)	0.653	0.650	(0.320–1.320)	0.233	0.787	(0.374–1.655)	0.528
Increased FM, yes	0.457	(0.225–0.925)	0.030	0.450	(0.221–0.916)	0.028	0.458	(0.223–0.938)	0.033	0.582	(0.275–1.233)	0.158
Muscle wasting, yes	0.967	(0.458–2.043)	0.930	0.880	(0.413–1.877)	0.741	0.868	(0.391–1.925)	0.728	0.837	(0.369–1.896)	0.669
eGFR, ml/min/1.73 m2	0.978	(0.964–0.993)	0.004				0.978	(0.962–0.993)	0.005	0.986	(0.969–1.004)	0.122
Diabetes, yes	1.560	(0.779–3.126)	0.210				1.190	(0.563–2.517)	0.649	1.108	(0.511–2.401)	0.794
NT-proBNP, pg/ml	1.884	(1.391–2.552)	< 0.001							1.488	(1.030–2.150)	0.034

*eGFR* estimated glomerular filtration ratio, *HR* hazard ratio, *CI* confidence interval

Increased percent body fat mass (increased FM) was defined as percent body fat > 25% in males and > 30% in females

Muscle wasting, i.e., reduction in skeletal muscle mass, was defined as appendicular skeletal muscle mass index < 7.0 kg/m^2^ in males and < 5.4 kg/m^2^ in females

## Discussion

The relationship between obesity and mortality is highly complex. The concept of the obesity paradox in a general population was derived from results of epidemiological studies showing a U- or J-shaped relationship between BMI-defined obesity and mortality [4–6]. However, this concept has been questioned by the results of a recent meta-analysis [19]. The meta-analysis used data for study subjects who had no smoking history (never smokers) and no chronic diseases at baseline and were followed up for at least 5 years. The results showed that both overweight (BMI, 25.0–29.9 kg/m^2^) and obesity (BMI > 30 kg/m^2^) were associated with increased all-cause mortality, while no significant increase in mortality was observed in subjects with lower BMIs [19]. The reason for the apparent discrepancy between the results of the meta-analysis and the results of earlier epidemiological studies is unclear, but difference in baseline characteristics of study subjects is a reasonable explanation. In other words, concurrent chronic diseases and/or smoking habits might have been involved in the increase in mortality in subjects with a lowest range BMIs in the epidemiological studies. In fact, this notion is supported by recent findings that reduction in BMI between the first and second hospitalizations for HF was associated with increased risks of subsequent hospitalizations and cardiovascular mortality [20]. Taken together, the results of the earlier studies suggest that chronic diseases underlying BMI reduction as well as obesity have detrimental effects on clinical outcomes, resulting in a U-shaped relationship between BMI and mortality. Whether BMI reduction associated with chronic disease is just a surrogate marker of severity of the disease or whether change in the body mass composition that occurs together with BMI reduction contributes to the mortality remains an important question.

A recent study by Aimo et al. examined the association of estimated percent body fat calculated by prediction equation with prognosis in HF patients to unveil the underlying mechanism of obesity paradox [21]. Although HF patients with the lowest tertile of estimated percent body fat had worse prognosis in that study, the results should be confirmed by using sophisticated assessment of percent body fat. In the present study, we examined compositions of the body by DEXA scans and their relationships with cardiac events during a 180-day follow-up period. Study subjects in the present study were relatively old and mostly classified as non-obese subjects by BMI criteria (Fig. 1). Interestingly, patients with increased FM had a significantly lower cardiac event rate during the follow-up period than did patients without increased FM, though blood biochemistry data were consistent with obesity (i.e., low HDL-cholesterol levels, high triglyceride levels, hyperinsulinemia) (Table 2, Fig. 2a). In contrast, there was no significant difference between cardiac event rates in patients with and those without muscle wasting (Fig. 2b). These results are consistent with the results of a recent study by Thomas et al. using bioelectrical impedance analysis (BIA) for determination of body fat mass in HF outpatients (*n* = 359) [22]. Thomas et al. reported that survival rate was significantly better in patients with body fat mass index ≥ the median (i.e., 8.2 kg/m^2^) than in patients with body fat index < the median [22]. Cox regression multivariate analysis indicated that body fat mass index, but not lean body mass index, was associated with improved survival rate. Despite multiple differences including differences in methods for fat mass determination (DEXA vs. BIA), cutoff levels of fat mass index and presence or absence of gender-specific cutoff levels, the results of the present study and the study by Thomas et al. support the notion that higher percent body fat, but not muscle mass, predicts a lower cardiac event rate in HF patients.

Adipose tissue serves as a critical regulator of systemic energy control [23, 24]. Under a condition in which there is an energy supply, adipose tissue stores excess energy in the form of lipid droplets. Conversely, adipose tissue supplies energy via lipid breakdown in response to a starved condition such as anorexia in HF-induced cachexia. However, the cachexia-induced fat depletion cannot be explained solely by compensatory utilization of adipose tissue for energy production since an increase in energy supply by parenteral nutrition does not reverse the cachexic state [25]. Chronic diseases including chronic HF provoke systemic inflammation induced by innate immune signaling [26, 27], leading to inappropriate degradation of adipose tissue [11, 28]. Catecholamine excess and hormone imbalance are aggravative factors in this process [11, 29]. Importantly, innate immune signaling in HF is upregulated in an HF severity-dependent manner [30–32], and recent studies have shown that inflammatory diseases as well as malnutrition and HF are closely associated with muscle wasting [33–36]. Albeit there being shared signal pathways leading to fat depletion and muscle wasting, reduction in body fat is not necessarily concordant with reduction in skeletal muscle mass in the clinical course of HF [11]. In the present study subjects, the proportion of patients with muscle wasting was higher than that of patients with no increased FM (67% vs. 42%). When patients were divided into two groups by ASMI, the group with muscle wasting had slightly smaller body fat mass (median, 27.1% vs. 30.0%). On the other hand, when patients were divided into two groups by a cutoff level of increased FM, there was no significant difference in skeletal muscle mass (median, 5.8 vs. 5.5 kg/m^2^) between the two groups. These findings suggest that distinct mechanisms are operative for preservation of adipose tissue and preservation of skeletal muscle mass, while skeletal muscle preservation is partly dependent on the adipose tissue mass. In fact, starvation induces depletion of both skeletal muscle and body fat, and moderate to severe obesity potentially induces muscle wasting by fat-induced systemic inflammation [37, 38]. Nevertheless, a significant association between cardiac events and preserved body fat mass (Fig. 2) supports the notion that a favorable energy storage/supply balance in adipose tissue contributes to prevention of cardiac events.

An increase in fat mass induces systemic inflammation including cytokine release, leading to pathological/functional myocardial dysfunction [38, 39]. In addition to the absolute mass of body fat, fat distribution is also tightly linked to the development and progression of metabolic diseases and HF [40, 41]. In a clinical setting, waist circumference has been applied as a surrogate marker of visceral fat mass, which is one of the diagnostic criteria of metabolic syndrome [42, 43]. The contribution of an increase in abdominal fat mass to the progression of heart failure was supported by the results of a recent study showing that abdominal obesity, defined as waist circumferences of > 102 cm in men and > 88 cm in women, in patients with HFpEF was significantly associated with an increased risk of all-cause mortality [40]. In addition, obesity and diseases characterized by chronic inflammation lead to epicardial fat accumulation [41]. There is evidence indicating that epicardial fat plays a detrimental role in the pathophysiology of HF. Epicardial fat thickness is closely associated with the extent of myocardial fibrosis, leading to myocardial dysfunction [44, 45]. Furthermore, accumulation of epicardial fat induces systemic inflammation, possibly contributing to further fat accumulation. Unfortunately, we could not include data for abdominal fat and data for epicardial fat in the present analyses because of technical limitations and the retrospective nature of the study.

**Fig. 4:**
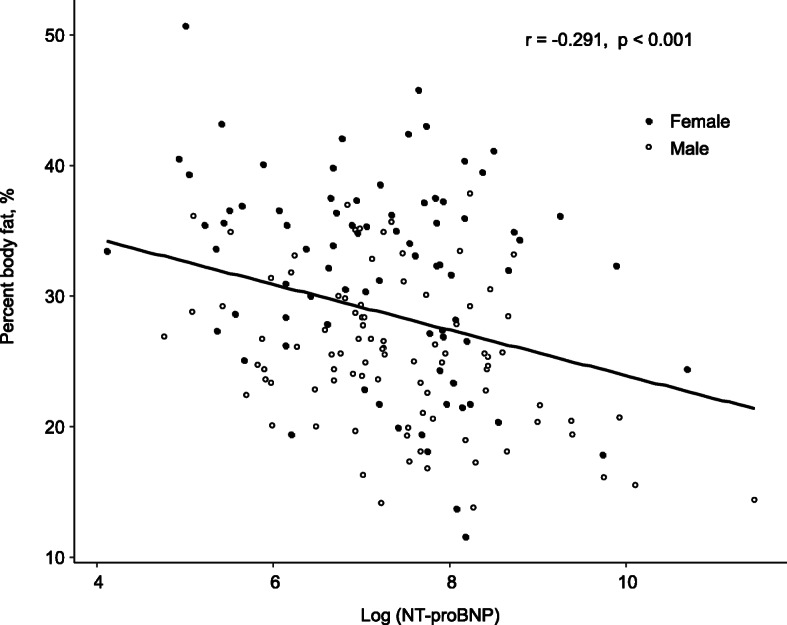
Association between percent body fat and NT-proBNP levels

Circulating natriuretic peptide (NP) level is an established marker of HF prognosis [46, 47]. Venous blood level of NP is positively correlated with the extent of myocardial stretch, which is usually reflected by increased filling pressure of the ventricle. In addition to such a ventricular pressure-dependent release of NP into the circulation, several regulatory mechanisms of circulating levels of NP have been reported. Levels of NPs, especially NT-proBNP, are elevated by renal failure since NPs are mainly cleared from circulating blood by renal excretion [48]. Hyperinsulinemia, frequently seen in obese patients, is associated with lower NT-proBNP levels [49, 50]. Circulating levels of proinflammatory cytokines such as TNF-α and IL-1β, which are pronouncedly increased in a cachexic state, increase production of brain natriuretic peptide from cardiomyocytes [51]. Interestingly, NP has recently been reported to significantly stimulate lipolysis [52]. Thus, there is the possibility that marked elevation of NP level in cachexic HF patients exerts a detrimental effect on HF by acceleration of lipolysis, resulting in reduction of body fat mass, though protective effects of NPs on cardiovascular and renal functions have already been characterized. In fact, the prognostic impact of low body fat mass in the present study was lost by the inclusion of NT-proBNP into the Cox proportional hazard model (Table 3). Furthermore, a negative correlation between percent body fat and NT-proBNP levels was found (Fig. 4), suggesting an interaction of body fat mass level and level of NT-proBNP in HF patients. On the other hand, the prognostic impact of increased FM was also lost by the inclusion of NYHA functional class into the Cox proportional hazard model (Supplementary Table 1), suggesting that HF severity overcomes percent body fat in the prediction of short-term prognosis in HF patients. These findings need to be confirmed in a large population-based study of HF.

Fat mass, muscle mass, and bone mineral content are reduced as cachexic conditions progress [13]. Our recent study reported that presence of osteoporosis assessed during hospital stay is an independent predictor of adverse events after discharge and fat mass index was closely associated with the extent of osteoporosis [53]. Result of a recent study showed that muscle wasting is an independent predictor of mortality in stable ambulatory HF patients [54]. Taken together with findings of the present study, muscle wasting may be an early marker for HF progression, whereas reduction in fat mass and bone mineral content may serve as a marker of cachexia. Importantly, adipose tissue supplies energy via lipid breakdown in response to a starved condition such as anorexia in HF-induced cachexia, which might play a role in reduced cardiac events at the advanced stage of HF. A potential therapeutic approach targeting cachexia including fat depletion is a nutritional intervention since results of our very recent study showed that energy intake during hospital stay is a strong predictor of all-cause mortality even in elderly HF patients [55]. However, an increase in energy supply by parenteral nutrition did not reverse the cachexic state [25]. Therefore, further analyses are needed to demonstrate the complex relationship between fat mass and prognosis.

There are limitations in the present study. First, since this study was a retrospective observational study using a small number of patients in a single center, there might have been selection bias in study subjects. Importantly, the present study might have insufficient statistical power for detection of effects of fat/muscle mass on cardiac events among the groups with different etiologies of heart failure, e.g., HFrEF vs. HFpEF, though results of post-host analyses showed that prognostic impact of increased FM tended to be found in patients with HFrEF and with heart failure with mid-range ejection fraction (Supplementary Figure 1–2 and Supplementary Table 2). Second, sarcopenic obesity, i.e. coexistence of obesity and sarcopenia, is frequently observed in HF patients with HFpEF, which is a risk factor of hospitalization and death [56, 57]. Small number of patients with HFpEF in the present study (45/198) might be responsible for loss of prognostic effect of sarcopenic obesity as shown by the data: presence of muscle wasting had no effects on cardiac event rates also in HF patients with increased FM (Fig. 3). In addition, muscle strength, a criterion of sarcopenia, was not analyzed in the present study, which might contribute to underestimation of well-known prognostic impact of sarcopenia in this study subjects [18, 58]. Therefore, further analyses are needed to demonstrate the impact of sarcopenic obesity on cardiac event rates in our study population. Third, the patients enrolled in the present study were patients who were admitted to our institute for diagnosis and/or treatment of HF. Patients who were admitted for acute decompensation heart failure were also included. Although assessment of body composition was performed after the relief of worsening HF, the findings in the present study may not be extrapolated to ambulatory HF patients. Finally, previous studies repeatedly showed race/region-dependent variation in body composition [59, 60]. Thus, the results of the present study may not necessarily be applicable to other ethnicities.

## Conclusions

Increased body fat mass, but not appendicular skeletal muscle mass, predicts a lower cardiac event rate after hospital discharge in non-obese HF patients.

## Supplementary Information

**Additional file 1.**

## Data Availability

The datasets generated and/or analyzed during the current study are not publicly available because a research agreement from all authors is required for data sharing, but are available from the corresponding author on reasonable request.

## Abbreviations

BMI: Body mass index
HF: Heart failure
LVEF: Left ventricular ejection fraction
FM: Fat mass
IQR: Interquartile range
HFrEF: Heart failure with reduced ejection fraction
HFpEF: Heart failure with preserved ejection fraction
DEXA: Dual-energy X-ray absorptiometry
NYHA: New York Heart Association
ASM: Appendicular skeletal muscle mass
ASMI: Appendicular skeletal muscle mass index
eGFR: Estimated glomerular filtration rate
NT-proBNP: pro B-type natriuretic peptide
CKD: Chronic kidney disease
NP: Natriuretic peptide
