# Supplementary material for: High percent body fat mass predicts lower risk of cardiac events in patients with heart failure: an explanation of the obesity paradox
Source: BMC Geriatr. 2021 Jan 6;21:16. doi: 10.1186/s12877-020-01950-9 (PMC7789382; doi:10.1186/s12877-020-01950-9)
Supplement: Supplementary file 1 — Additional file 1. [file 12877_2020_1950_MOESM1_ESM.docx]

**Online supplement**

High percent body fat mass predicts lower risk of cardiac events in patients with heart failure: an explanation of the obesity paradox

Katsuhiko Ohori, MD, PhD^1,2^, Toshiyuki Yano, MD, PhD^1#^, Satoshi Katano, PT, MS^3^, Hidemichi Kouzu, MD, PhD^1^, Suguru Honma, PT^3^, Kanako Shimomura, PT^3^, Takuya Inoue, PT^3^, Yuhei Takamura, PT^3^, Ryohei Nagaoka, PT^3^, Masayuki Koyama, MD, PhD^1,4^, Nobutaka Nagano, MD^1^, Takefumi Fujito, MD^1^, Ryo Nishikawa, MD^1^, Tomoyuki Ishigo, Ph^5^, Ayako Watanabe, RN^6^, Akiyoshi Hashimoto, MD, PhD^1,7^, Tetsuji Miura, MD, PhD^1^

^1^Department of Cardiovascular, Renal and Metabolic Medicine, Sapporo Medical University School of Medicine, Sapporo, Japan.

^2^Department of Cardiology, Hokkaido Cardiovascular Hospital, Sapporo, Japan.

^3^Division of Rehabilitation, Sapporo Medical University Hospital, Sapporo, Japan.

^4^Department of Public Health, Sapporo Medical University School of Medicine, Sapporo, Japan.

^5^Division of Hospital Pharmacy, Sapporo Medical University Hospital, Sapporo, Japan.

^6^Division of Nursing, Sapporo Medical University Hospital, Sapporo, Japan.

^7^Division of Health Care Administration and Management, Sapporo Medical University School of Medicine, Sapporo, Japan.

Short title: Percent body fat and cardiac event in heart failure

**Address for correspondence**

Toshiyuki Yano, MD, PhD, FESC, FJCS

Department of Cardiovascular, Renal, and Metabolic Medicine

Sapporo Medical University School of Medicine

South-1, West-16, Chuo-ku

Sapporo 060-8543, Japan

Phone: +81-11-611-2111, ext. 3225

Fax: +81-11-644-7958

Email: [tyano@sapmed.ac.jp](mailto:miura@sapmed.ac.jp)

**
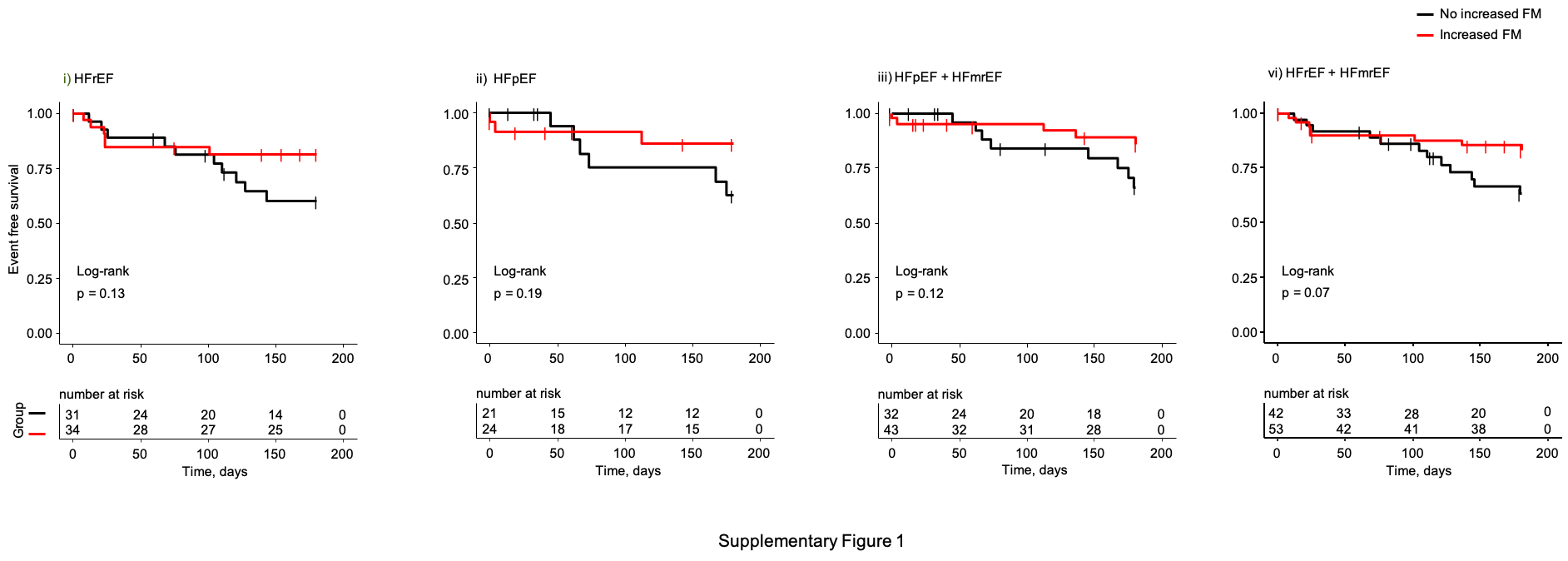
**

**
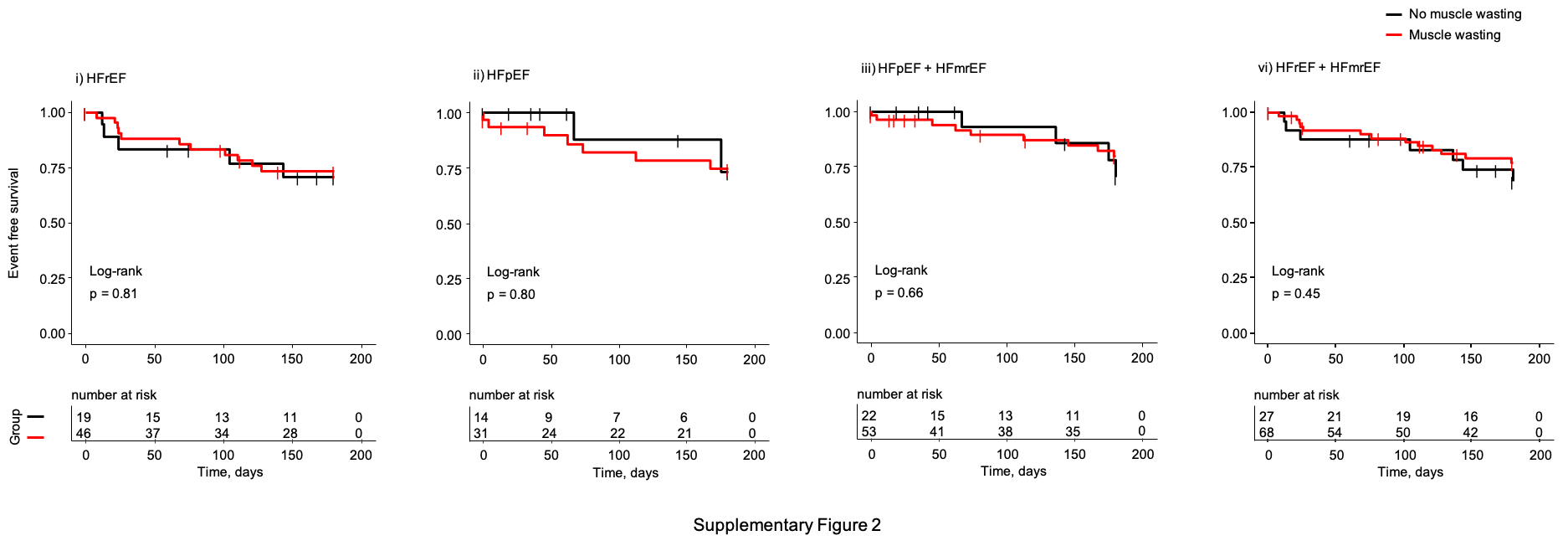
**

**Supplementary Figure legends**

**Supplementary Figure 1** Kaplan-Meier event-free survival curves in heart failure patients stratified by LVEF categories: increase FM vs. no increased FM

Patients were divided into three groups according to LVEF categories, i.e., heart failure with reduced ejection fraction (HFrEF), left ventricular ejection fraction (LVEF)<40%; heart failure with mid-range ejection fraction (HFmrEF), 40%≦LVEF<50%; heart failure with preserved ejection fraction (HFpEF), 50≦LVEF. Increased percent body fat mass (increased FM) was defined as dual-energy X-ray absorptiometry-measured percent body fat mass > 25% in males and > 30% in females.

**Supplementary Figure 2** Kaplan-Meier event-free survival curves in heart failure patients stratified by LVEF categories: muscle wasting vs. no muscle wasting

Patients were divided into three groups according to LVEF categories, i.e., heart failure with reduced ejection fraction (HFrEF), left ventricular ejection fraction (LVEF)<40%; heart failure with mid-range ejection fraction (HFmrEF), 40%≦LVEF<50%; heart failure with preserved ejection fraction (HFpEF), 50≦LVEF. Muscle wasting was defined by the cut-off values of appendicular skeletal muscle mass index: <7.00 kg/m^2^ in males and <5.40 kg/m^2^ in females.

Supplementary Table 1 Multivariate analyses by Cox-proportional hazards model

| Parameters | Univariate model | | | Multivariate model | | | | | |
| --- | --- | --- | --- | --- | --- | --- | --- | --- | --- |
|  |  |  |  | model 4 | | | model 5 | | |
|  | HR | 95% CI | p value | HR | 95% CI | p value | HR | 95% CI | p value |
| Age, yrs | 1.003 | (0.978 - 1.029) | 0.808 | 0.998 | (0.968 - 1.029) | 0.909 | 0.99 | (0.963 - 1.019) | 0.507 |
| Gender, male | 0.848 | (0.424 - 1.697) | 0.641 | 0.651 | (0.320 - 1.322) | 0.235 | 0.809 | (0.392 - 1.669) | 0.566 |
| Increased FM, yes | 0.457 | (0.225 - 0.925) | 0.030 | 0.475 | (0.231 - 0.976) | 0.043 | 0.546 | (0.266 - 1.123) | 0.100 |
| Muscle wasting, yes | 0.967 | (0.458 - 2.043) | 0.930 | 0.905 | (0.403 - 2.036) | 0.810 | 0.657 | (0.287 - 1.506) | 0.321 |
| eGFR, ml/min/1.73m^2^ | 0.978 | (0.964 - 0.993) | 0.004 | 0.980 | (0.964 - 0.997) | 0.018 | 0.979 | (0.964 - 0.994) | 0.007 |
| Diabetes, yes | 1.560 | (0.779 - 3.126) | 0.210 | 1.098 | (0.508 - 2.376) | 0.812 | 1.060 | (0.493 - 2.277) | 0.881 |
| NT-proBNP, pg/ml | 1.884 | (1.391 - 2.552) | <0.001 |  |  |  |  |  |  |
| LVEF, % | 0.978 | (0.957 – 0.999) | 0.045 | 0.985 | (0.961 - 1.009) | 0.207 |  |  |  |
| NYHA-FC III, yes | 3.826 | (1.769 - 8.276) | <0.001 |  |  |  | 3.811 | (1.686 - 8.616) | 0.001 |

eGFR, estimated glomerular filtration ratio; HR, hazard ratio; CI, confidence interval

Increased percent body fat mass (increased FM) was defined as percent body fat > 25% in males and >30% in females.

Muscle wasting, i.e., reduction in skeletal muscle mass, was defined as appendicular skeletal muscle mass index <7.0 kg/m^2^ in males and <5.4 kg/m^2^ in females.

Supplementary Table 2 Patients characteristics stratified by LVEF categories

|  | HFrEF | HFmrEF | HFpEF | Valvular heart disease | p value |
| --- | --- | --- | --- | --- | --- |
| N | 65 | 48 | 27 | 58 |  |
| Age, yrs. | 71 [58 - 77] | 71 [64 - 78] | 79 [71 - 85] | 82 [76 - 86] | <0.001 |
| Male, n (%) | 44 (67.7) | 29 (60.4) | 12 (44.4) | 17 (29.3) | <0.001 |
| Height, cm | 161.7 (11.1) | 160.9 (8.2) | 156.4 (8.6) | 151.5 (7.8) | <0.001 |
| Weight, kg | 55.3 [46.6 - 68.9] | 58.0 [48.3 – 65.9] | 51.8 [43.9 - 61.6] | 50.6 [46.4 - 57.3] | 0.051 |
| BMI, kg/m^2^ | 21.7 [19.4 - 24.0] | 22.2 [19.2 - 24.6] | 20.6 [19.1 - 23.7] | 22.3 [20.7 - 24.3] | 0.436 |
| NYHA-FC III, n (%) | 34 (52.3) | 19 (39.6) | 13 (48.1) | 21 (36.2) | 0.282 |
| LVEF, % | 28.9 [24.6 - 32.8] | 47.5 [44.5 - 54.0] | 68.0 [64.5 - 71.1] | 62.1 [55.7 - 65.8] | <0.001 |
| eGFR, ml/min/1.73cm^2^ | 43.9 [24.6 - 62.2] | 57.7 [42.9 - 68.1] | 41.1 [25.7 - 72.9] | 50.3 [39.7 - 66.8] | 0.053 |
| NT-proBNP, pg/ml | 2212 [1023 - 5443] | 1424 [938 - 3278] | 1353 [557 - 2080.] | 1269 [312 - 2848] | 0.003 |
|  |  |  |  |  |  |
| **Comorbidity** |  |  |  |  |  |
| Hypertension | 44 (67.7) | 28 (59.6) | 19 (70.4) | 47 (81.0) | 0.086 |
| Dyslipidemia | 37 (56.9) | 19 (40.4) | 12 (44.4) | 35 (60.3) | 0.120 |
| Diabetes | 32 (49.2) | 14 (29.8) | 12 (44.4) | 18 (31.0) | 0.082 |
| Chronic kidney disease | 34 (52.3) | 18 (38.3) | 15 (55.6) | 31 (53.4) | 0.292 |
|  |  |  |  |  |  |
| **Medication** |  |  |  |  |  |
| ACE-I or ARB | 36 (55.4) | 26 (54.2) | 9 (33.3) | 20 (34.5) | 0.038 |
| Beta blocker | 51 (78.5) | 38 (80.9) | 16 (59.3) | 29 (50.0) | 0.001 |
| Loop diuretics | 51 (78.5) | 30 (63.8) | 13 (48.1) | 28 (48.3) | 0.003 |
| MRA | 37 (56.9) | 25 (53.2) | 11 (40.7) | 19 (32.8) | 0.042 |
|  |  |  |  |  |  |
| **Etiology** |  |  |  |  | <0.001 |
| Cardiomyopathy | 43 (66.2) | 22 (45.8) | 8 (29.6) | 0 (0.0) | |
| Valvular heart disease | 0 (0.0) | 0 (0.0) | 0 (0.0) | 58 (100.0) | |
| Ischemic heart disease | 18 (27.7) | 15 (31.9) | 5 (18.5) | 0 (0.0) | |
| Others | 4 (6.2) | 11 (23.4) | 14 (51.9) | 0 (0.0) | |
|  |  |  |  |  | |
| ASMI, kg/m^2^ | 5.78 [4.99 - 6.64] | 5.55 [4.94 - 6.58] | 5.29 [4.79 - 5.97] | 5.70 [5.02 - 6.36] | 0.475 |
| Muscle wasting, n (%) | 46 (70.8) | 33 (68.8) | 20 (74.1) | 34 (58.6) | 0.401 |
| PBF, % | 26.7 [21.7 - 31.4] | 28.3 [23.4 - 35.0] | 27.1 [23.4 - 33.8] | 30.5 [25.5 - 34.9] | 0.133 |
| Increased FM, n (%) | 34 (52.3) | 28 (58.3) | 15 (55.6) | 37 (63.8) | 0.634 |

BMI, body mass index; NYHA-FC, New York heart association-functional class; LVEF, left ventricular ejection fraction;

eGFR, estimated glomerular filtration ratio; ACE-I, angiotensin-converting-enzyme inhibitor; ARB, angiotensin II receptor blocker;

MRA, mineralocorticoid receptor antagonist; ASMI, appendicular skeletal muscle mass index; PBF, percent body fat

Muscle wasting, i.e., reduction in skeletal muscle mass, was defined as appendicular skeletal muscle mass index <7.0 kg/m^2^ in males and <5.4 kg/m^2^ in females.

Increased percent body fat mass (increased FM) was defined as percent body fat > 25% in males and >30% in females.
